# Supplementary material for: Formulation of a Gastroretentive In Situ Oral Gel Containing Metformin HCl Based on DoE
Source: Pharmaceutics. 2022 Aug 25;14(9):1777. doi: 10.3390/pharmaceutics14091777 (PMC9504191; doi:10.3390/pharmaceutics14091777)
Supplement: Supplementary file 1 [file pharmaceutics-14-01777-s001.zip › pharmaceutics-1851215-supplementary.pdf]

**Table S1. The floating time for formulation E1**

| Batch No. | lag time (s) | s.d. | relative s.d. (%) | duration |
|---|---|---|---|---|
| E1-01 | 48 | 3 | 6 | > 12 h |
| E1-02 | 71 | 4 | 5 | > 12 h |
| E1-03 | 83 | 12 | 14 | > 12 h |
| E1-04 | 110 | 6 | 5 | > 12 h |
| E1-05 | 54 | 6 | 11 | > 12 h |
| E1-06 | 57 | 9 | 15 | > 12 h |
| E1-07 | 59 | 10 | 18 | > 12 h |
| E1-08 | 50 | 8 | 17 | > 12 h |
| E1-09 | 52 | 6 | 12 | > 12 h |

**Table S2. The viscosity of formulation E1**

| Batch No. | Viscosity (mPa·s) | s.d. | relative s.d. (%) |
|---|---|---|---|
| E1-01 | 304.8 | 2.5 | 0.8 |
| E1-02 | 300.4 | 2.1 | 0.7 |
| E1-03 | 248.6 | 4.8 | 2 |
| E1-04 | 406.8 | 4.3 | 1.1 |
| E1-05 | 513.6 | 2.4 | 0.5 |
| E1-06 | 548 | 13.2 | 2.4 |
| E1-07 | 688 | 229.3 | 33.3 |
| E1-08 | 1318.3 | 9.1 | 0.7 |
| E1-09 | 1283.3 | 24.1 | 1.9 |

**Table S3. The pH values of formulation E1**

| Batch No. | pH | s.d. |
|---|---|---|
| E1-01 | 8.79 | 0 |
| E1-02 | 8.82 | 0.01 |
| E1-03 | 9.2 | 0.01 |
| E1-04 | 9.2 | 0.01 |
| E1-05 | 9.04 | 0 |
| E1-06 | 9 | 0.01 |
| E1-07 | 9.16 | 0.01 |
| E1-08 | 9.16 | 0 |
| E1-09 | 9.17 | 0.01 |

**Table S4. The floating time for formulation E2**

| Batch No. | lag time (s) | s.d. | relative s.d. (%) | duration |
|---|---|---|---|---|
| E2-01 | 51 | 12 | 23 | > 12 h |
| E2-02 | 51 | 9 | 17 | > 12 h |
| E2-03 | 56 | 10 | 18 | > 12 h |
| E2-04 | 56 | 7 | 12 | > 12 h |
| E2-05 | 44 | 2 | 3 | > 12 h |
| E2-06 | 67 | 12 | 17 | > 12 h |
| E2-07 | 75 | 5 | 6 | > 12 h |
| E2-08 | 80 | 6 | 7 | > 12 h |
| E2-09 | 55 | 10 | 18 | > 12 h |
| E2-10 | 43 | 8 | 19 | > 12 h |
| E2-11 | 59 | 4 | 6 | > 12 h |
| E2-12 | 46 | 10 | 22 | > 12 h |
| E2-13 | 70 | 5 | 7 | > 12 h |
| E2-14 | 74 | 11 | 15 | > 12 h |
| E2-15 | 61 | 3 | 4 | > 12 h |
| E2-16 | 57 | 3 | 6 | > 12 h |
| E2-17 | 62 | 4 | 6 | > 12 h |

**Table S5. The viscosity of formulation E2**

| Batch No. | Viscosity (mPa·s) | s.d. | relative s.d. (%) |
|---|---|---|---|
| E2-01 | 1721 | 7 | 0.4 |
| E2-02 | 3960 | 12 | 0.3 |
| E2-03 | 4320 | 24 | 0.6 |
| E2-04 | 7888 | 42 | 0.5 |
| E2-05 | 2448 | 21 | 0.8 |
| E2-06 | 4472 | 25 | 0.6 |
| E2-07 | 3624 | 12 | 0.3 |
| E2-08 | 7388 | 37 | 0.5 |
| E2-09 | 1715 | 5 | 0.3 |
| E2-10 | 6400 | 91 | 1.4 |
| E2-11 | 1669 | 17 | 1 |
| E2-12 | 6472 | 90 | 1.4 |
| E2-13 | 4136 | 14 | 0.3 |
| E2-14 | 4072 | 14 | 0.3 |
| E2-15 | 4384 | 18 | 0.4 |
| E2-16 | 4716 | 43 | 0.9 |
| E2-17 | 3852 | 43 | 1.1 |

**Table S6. The pH values of formulation E2**

| Batch No. | pH | s.d. |
|---|---|---|
| E2-01 | 9.16 | 0 |
| E2-02 | 9.08 | 0 |
| E2-03 | 9.06 | 0.01 |
| E2-04 | 9.02 | 0.01 |
| E2-05 | 8.52 | 0.04 |
| E2-06 | 8.8 | 0.04 |
| E2-07 | 8.57 | 0.01 |
| E2-08 | 8.97 | 0.01 |
| E2-09 | 8.85 | 0.01 |
| E2-10 | 8.84 | 0.01 |
| E2-11 | 8.54 | 0.01 |
| E2-12 | 8.73 | 0.01 |
| E2-13 | 8.57 | 0.01 |
| E2-14 | 8.81 | 0.01 |
| E2-15 | 8.52 | 0.01 |
| E2-16 | 8.77 | 0.01 |
| E2-17 | 8.59 | 0.01 |
